# Mucopolysaccharidosis (MPS): Review of the literature and case series of five pediatric dental patients

**DOI:** 10.1002/ccr3.3885

**Published:** 2021-02-04

**Authors:** Lorna Hirst, Suhaym Mubeen, Gehan Abou‐Ameira, Anupam Chakrapani

**Affiliations:** ^1^ Dental and Maxillofacial Department Great Ormond Street Hospital London UK; ^2^ Metabolic Department Great Ormond Street Hospital London UK

**Keywords:** dentistry, metabolic disorders, pediatrics

## Abstract

Patients with MPS present with a plethora of dental manifestations, accompanying behavioral difficulties and medical comorbidities which often dictate the need for treatment in specialist centers. Prevention is therefore mandatory in this high‐risk group.

## Graphical abstract

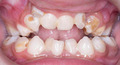

## 1 INTRODUCTION

The purpose of this article is to present the heterogeneity of dental manifestations and medical management of five pediatric patients with mucopolysaccharidosis; MPS I‐H (Hurler Syndrome), MPS II (Hunter Syndrome), MPS IIIA and MPS IIIB (Sanfilippo Syndrome), and MPS IVA (Morquio Syndrome) who were referred to the Dental Department at Great Ormond Street Hospital for Children, a tertiary referral hospital. Mucopolysaccharidosis (MPS) is a lysosomal storage disorder associated with significant morbidity and mortality. The seven distinct types of MPS present with dental anomalies, deviations in eruption, malocclusions, TMJ pathology, and increased risk of caries and periodontal disease with varying orthodontic and pediatric implications. Accompanying behavioral difficulties provide a characteristic feature of certain types of MPS and complicate treatment provision under local anesthesia. Medical comorbidities often dictate the need for treatment in specialist centers that are conducive to multidisciplinary management. This article reviews the relevant published literature on mucopolysaccharidosis, emphasizing the key concepts for optimal dental management.

Mucopolysaccharidosis (MPS) is a rare and heterogenous type of Inborn Error of Metabolism (IEM). Specifically, MPS is a lysosomal storage disorder characterized by the intralysosomal storage of glycosaminoglycans (GAGs).^1^ MPS is a chronic metabolic disorder, displaying significant morbidity and mortality due to the progressive multisystemic accumulation of GAGs.^2^ Seven distinct types of MPS exist (Type I, II, III, IV, VI, VII, IX) with further classification determined by which of the eleven enzymes responsible for the breakdown of GAGs is deficient.^1^ Heterogeneity across the scope of mucopolysaccharidosis results in a myriad of dental manifestations relevant to the pediatric dentist and orthodontist. The range of abnormalities is vast and broadly encompasses dental anomalies, deviations in eruption, TMJ pathology, malocclusion, and increased caries and periodontal disease.^1^, ^3^ Dental presentations amongt the distinct types of MPS is variable and interrelated, creating a diagnostic challenge even amongst astute clinicians.

This article aims to review the published literature on the dental phenotype of mucopolysaccharidosis and present five pediatric patients with diagnoses of MPS I‐H (Hurler Syndrome), MPS II (Hunter Syndrome), MPS IIIA and MPS IIIB (Sanfilippo Syndrome), and MPS IVA (Morquio Syndrome).

## 2 CASE DESCRIPTIONS

### 2.1 Case One: Mucopolysaccharidosis type I (Hurler Syndrome)

The first case describes a six‐year‐old female with mucopolysaccharidosis type I (Hurler Syndrome) who was referred to the dental department by her metabolic consultant. She received a bone marrow transplant at age 2 years, 2 months. Medically, she also had cleft lip (repaired at 36 weeks), obstructive sleep apnea (hypopnea syndrome), adenotonsillectomy (completed at 23 months), dysplastic mitral valve and regurgitation, kyphoscoliosis, corneal clouding, mixed bilateral hearing loss, and behavioral difficulties.

She was initially referred to clinical genetics due to a family history of cleft lip and palate. Clinically, it was observed that she had coarse facial features, developmental delay with associated nystagmus and hepatomegaly, and was subsequently referred to the metabolic department. It was noted that she had absence of alpha‐iduronidase in the white blood cells with increased urinary glycosaminoglycans. Genetically, she had a compound heterozygote mutation (c1045G < T, c1469T) in the alpha‐IDUA gene.

**FIGURE 1:**
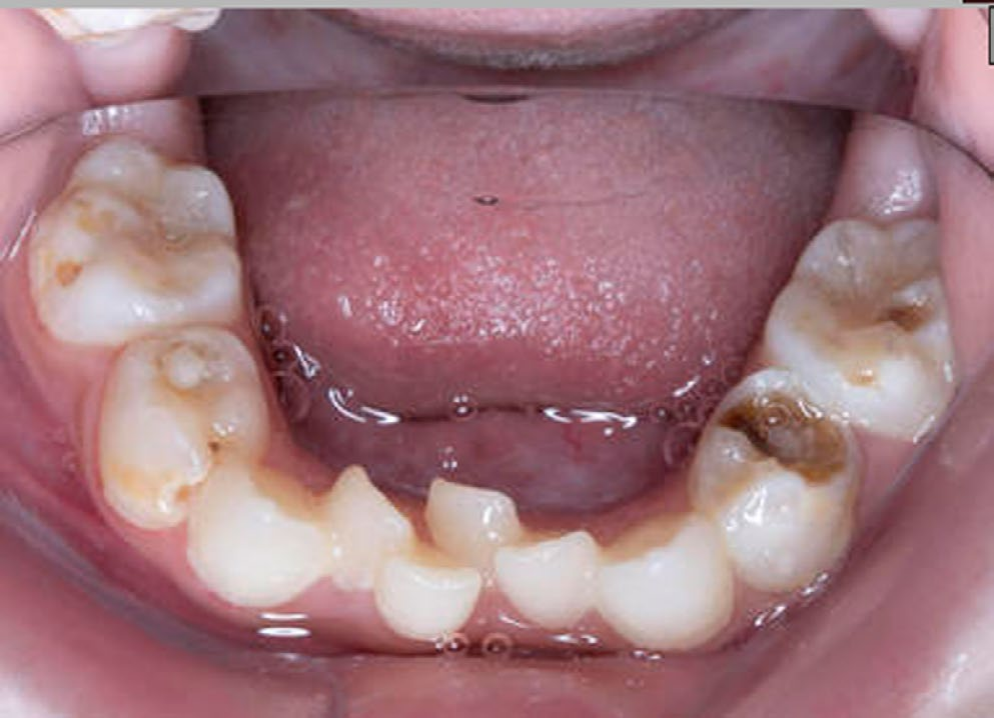
Intraoral image of the mandibular arch of case one (MPS IH) showing gross caries

**FIGURE 2:**
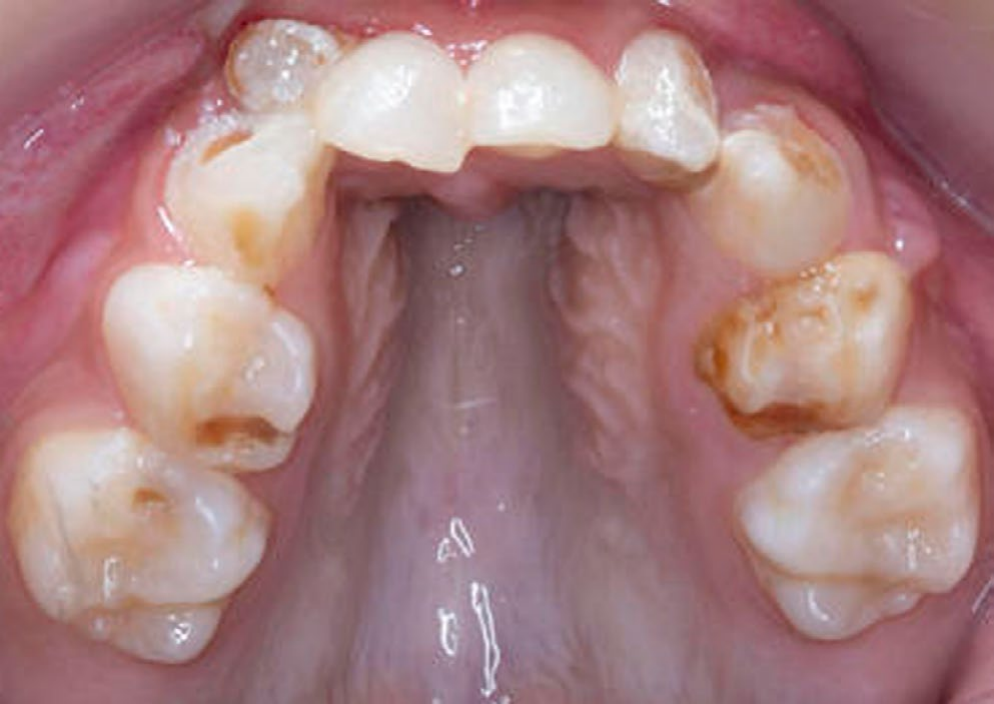
Intraoral image of the maxillary arch of case one (MPS IH) showing a high arched palate and gross caries

**FIGURE 3:**
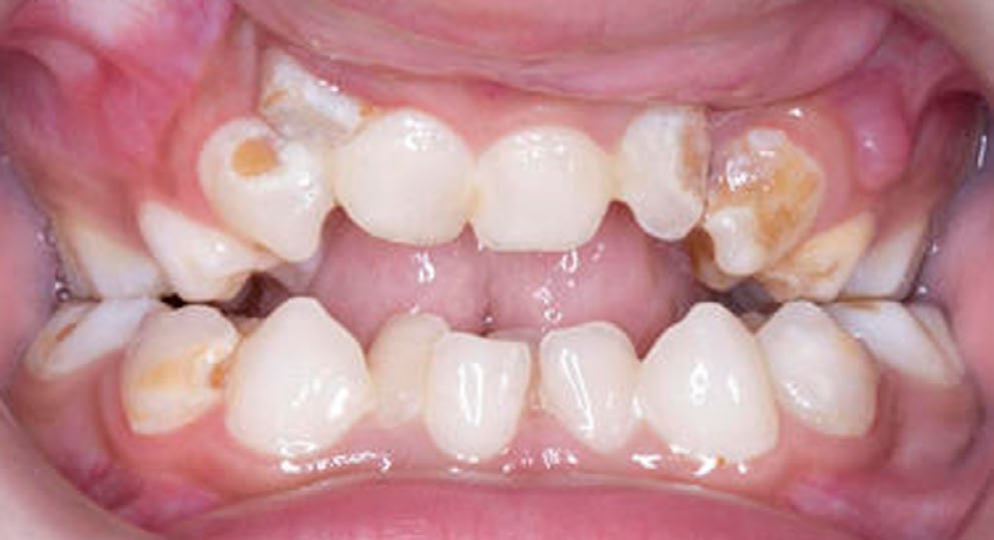
Intraoral image of case one (MPS IH) showing an anterior open bite and gross caries

She was referred to the pediatric dental department aged six years, seven months with odontogenic pain. Intraorally, she had a high arched palate, anterior open bite, crowding, and generalized gross caries and plaque induced gingivitis (Figures 1, 2, 3). She received extractions of all primary maxillary teeth, mandibular primary molars, and mandibular left central incisor, which was lingually displaced, in addition to 22 600 ppm fluoride varnish. The treatment was completed under general anesthesia aged six years, nine months.

### 2.2 Case Two: Mucopolysaccharidosis type II (Hunter Syndrome)

**FIGURE 4:**
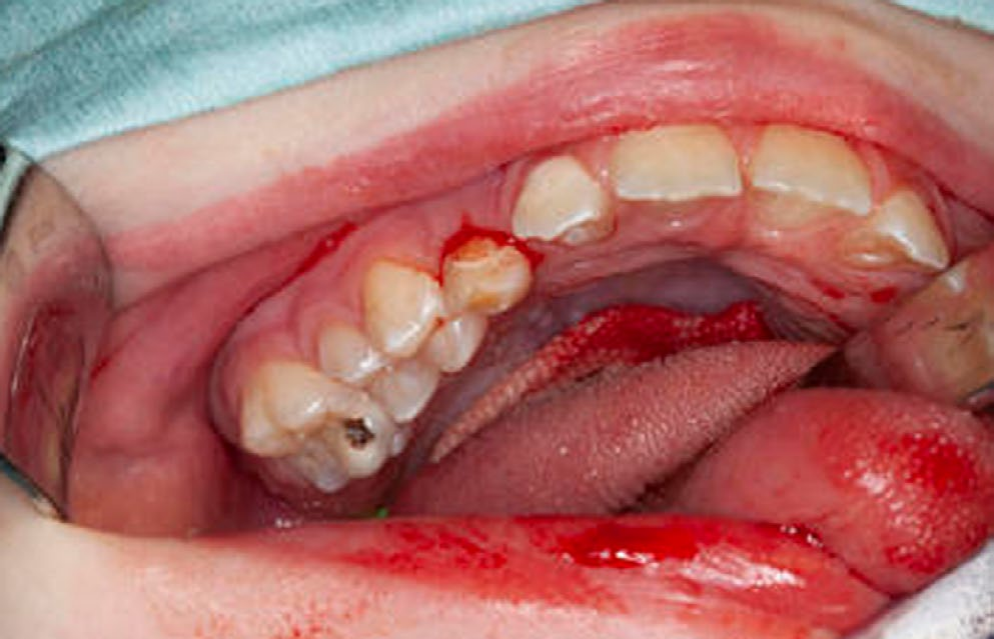
Intraoral view showing unrestorable caries in UR6 and URC

**FIGURE 5:**
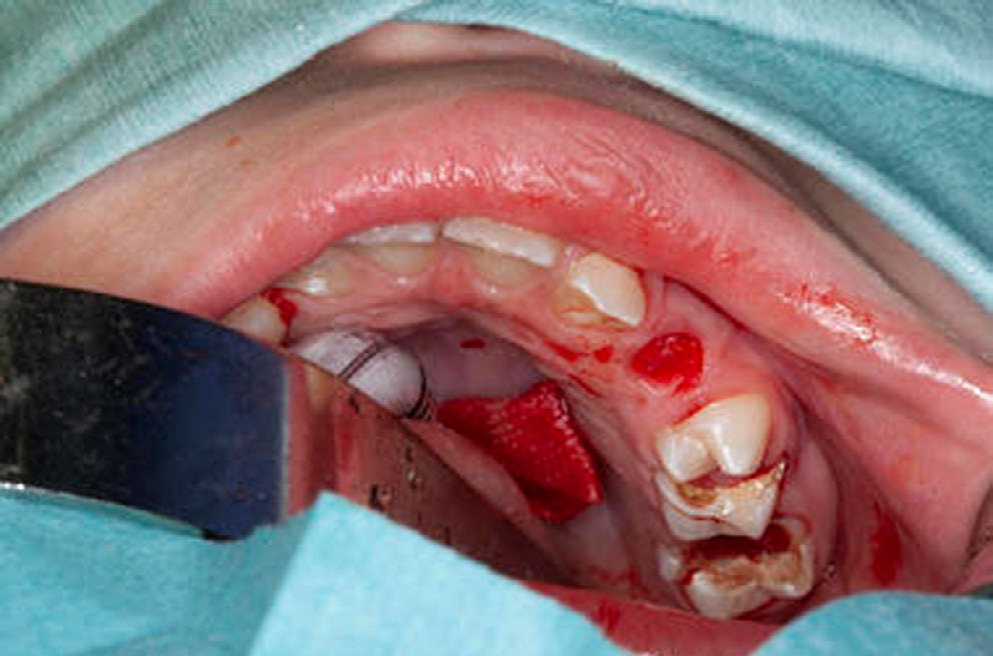
Intraoral view showing unrestorable caries in the ULE and UL6

The second case describes an eight‐year‐old male with mucopolysaccharidosis type II (Hunter Syndrome). His metabolic management was weekly intravenous idursulfase. Additionally, he had autistic spectrum disorder and behavioral difficulties. He was referred to the dental department by his metabolic consultant for odontogenic pain. Upon questioning, he had a highly cariogenic diet and was regularly drinking honey mixed with water. On examination, mandibular second primary molars and first permanent molars were grossly carious. The maxillary first permanent molars and second primary molars also had caries and maxillary primary canines were mobile (Figures 4, 5). He received dental treatment under general anesthesia, where an attempt was made to restore the mandibular right first permanent molar (LR6). The caries, however, was into the pulp and therefore, a decision was made to extract. Fissure sealants were placed on the right permanent premolars (UR5, UR4, LR5, LR4) and the maxillary left first premolar (UL4). The other premolars were partially erupted. All first permanent molars were extracted due to gross caries (UR6, UL6, LR6, LL6) in addition to maxillary primary second molars and canines (URE, URC, ULC, ULE). Regarding prevention, 22 600 ppm fluoride varnish was applied in addition to dietary counselling and oral hygiene instruction.

### 2.3 Case Three: Mucopolysaccharidosis type IIIA (Sanfilippo Syndrome)

The third patient describes a twelve‐year‐old female who was referred internally from her metabolic consultant concerning an acute apical abscess. Medically, she had mucopolysaccharidosis IIIA (Sanfilippo Syndrome). Medical comorbidities included gastroesophageal reflux, megarectum, chronic constipation, reduced visual acuity, and behavioral and learning difficulties. Medication included lansoprazole, macrogol, and aripiprazole. She was under the care of a metabolic dietician who prescribed frequent fruit smoothies and fruit yoghurt consumption to aid bowel movements.

**FIGURE 6:**
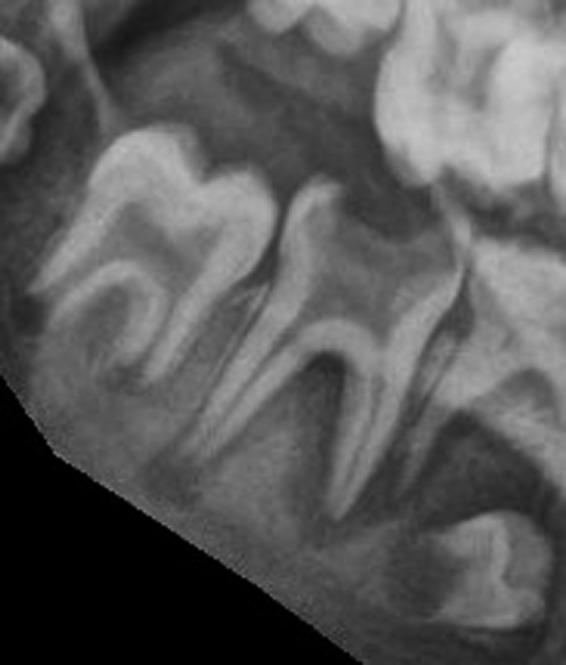
Acute apical abscess of left mandibular first permanent molar (LL6) and second primary molar (LLE)

Comprehensive oral examination was constrained by difficulties in cooperation. Fortuitously, compliance permitted lateral oblique radiography (Figure 6) and a diagnosis of acute apical abscess of the left mandibular first permanent molar (LL6) and second primary molar (LLE) and a retained primary first molar (LLD) was confirmed. Due to the extent of the infection, extraction was deemed the treatment of choice. An orthodontic opinion was obtained regarding the horizontal inclination of the left mandibular second premolar (LL5) and the benefits of extracting the maxillary left first permanent molar (UL6) as a compensating extraction. Insufficient cooperation mandated general anesthesia for a detailed examination, fissure sealants of the right maxillary and mandibular first permanent molars (UR6, LR6) and extraction of left maxillary and mandibular first permanent molars (UL6, LL6), left mandibular primary first and second molars (LLE, LLD), and fluoride varnish (22 600 ppm) application.

### 2.4 Case Four: Mucopolysaccharidosis type IIIB (Sanfilippo Syndrome)

Case four describes a seven‐year‐old male with mucopolysaccharidosis type IIIB (Sanfilippo Syndrome). Medical comorbidities included mitral and aortic valve dysplasia, intellectual disability, behavioral challenges, abnormal gait, and impaired hearing. On clinical examination, he had coarse facial features and hirsutism.

**FIGURE 7:**
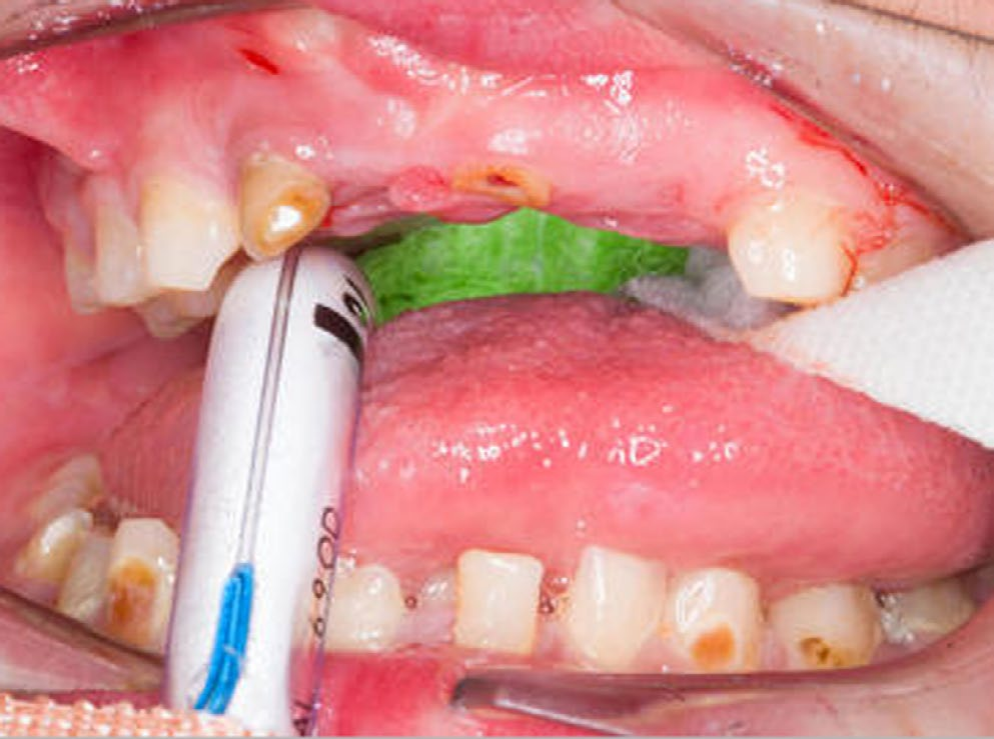
Intraoral views demonstrating gross caries

**FIGURE 8:**
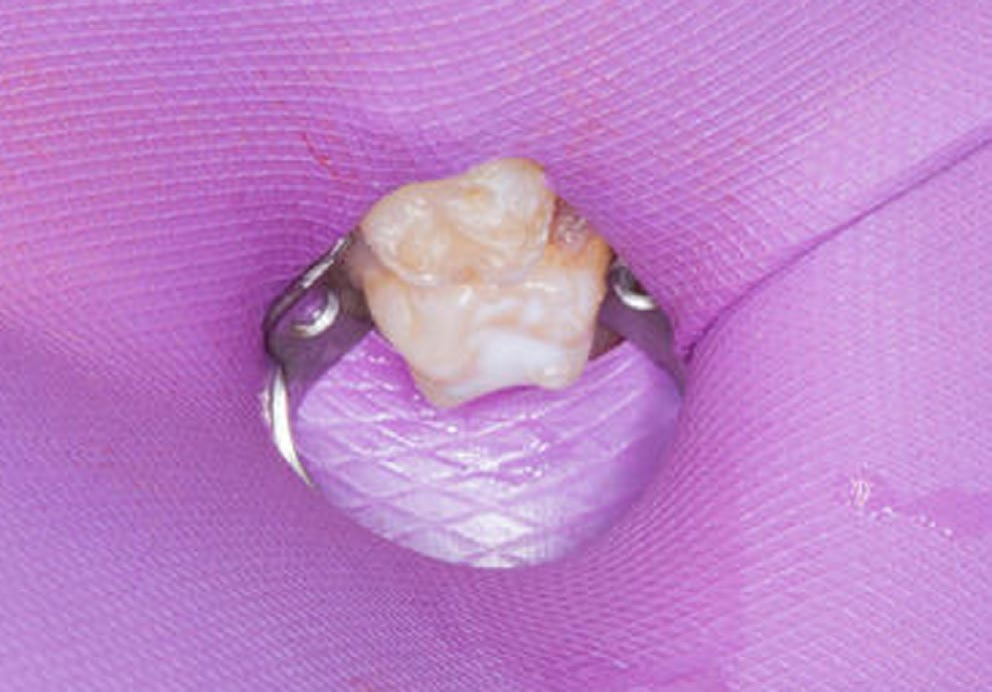
Composite restoration of the ULE

He was referred to the dental department for gross caries (Figure 7) and received comprehensive dental treatment under general anesthesia. This included composite restoration and fissure sealant provision of the maxillary left second primary molar (ULE) (Figure 8) and multiple extractions; mandibular primary molars and canines (LRE, LRD, LRC, LLC, LLD, LLE), mandibular left primary central incisor (LLA), maxillary right primary lateral incisor (URB), and left central incisor (ULA). He also received 22,600ppm fluoride application.

### 2.5 Case Five: Mucopolysaccharidosis type IVA (Morquio Syndrome)

The fifth case describes a twelve‐year‐old female referred to the dental department internally from the metabolic department. Medically, she had Mucopolysaccharidosis type IVA (Morquio Syndrome (MPS IVA)), severe obstructive sleep apnea requiring nocturnal CPAP, bilateral conductive and sensorineural hearing loss, bilateral corneal clouding, left ventricular hypertrophy, congenital bilateral talipes equinovarus (surgically corrected), genu valgum (surgically corrected), atlanto‐axial instability (awaiting surgical stabilisation), and short stature. Her MPS IVA was managed with Vimizim, an enzyme replacement therapy (ERT).

**FIGURE 9:**
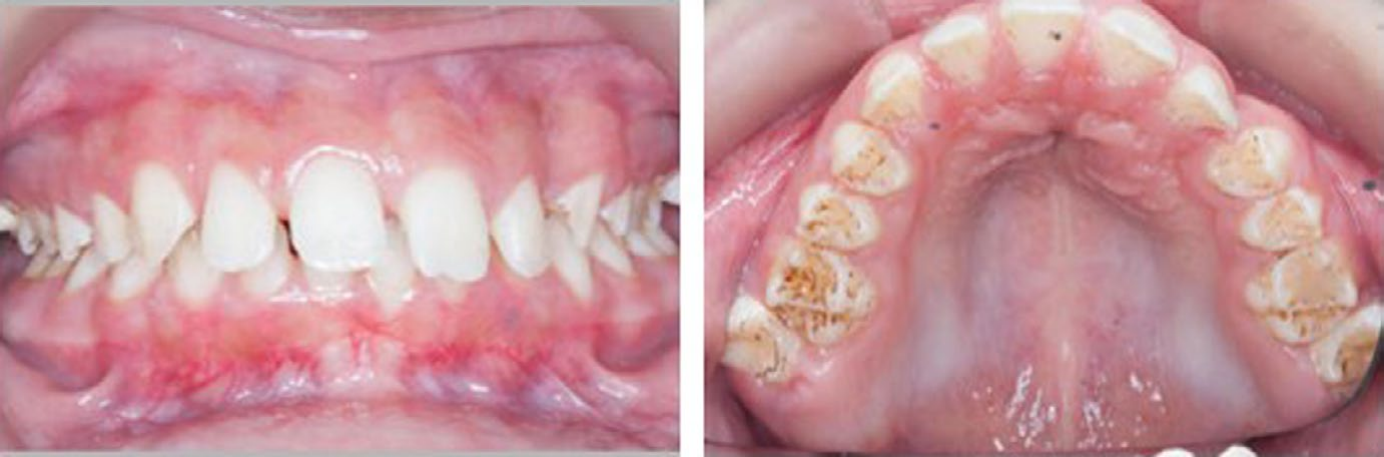
Intraoral views showing microdontia, maxillary anterior spacing, and hypoplastic enamel

**FIGURE 10:**
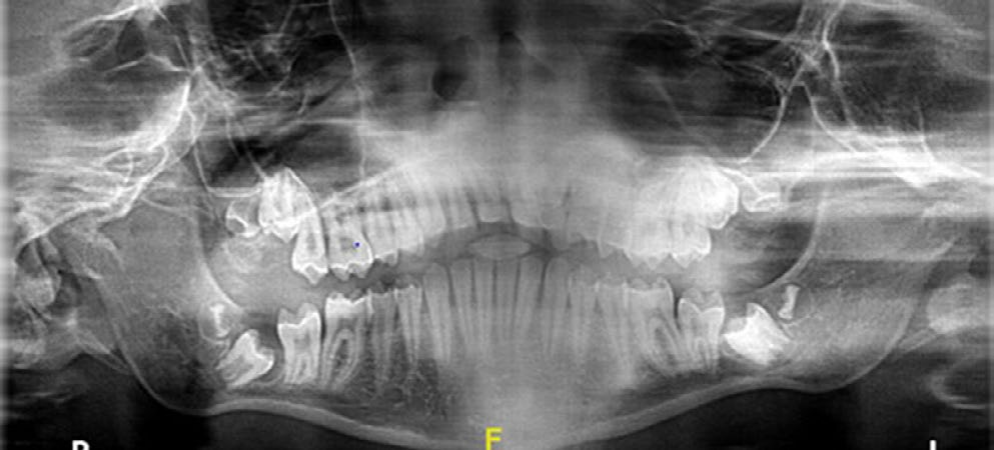
Orthopantomogram showing canine ectopy (UL3) and 4 supernumerary teeth developing distal to the third permanent molars

The patient was referred to the orthodontic department regarding canine ectopy and supernumerary teeth. A retrognathic mandible and proclined maxillary incisors contributed to an increased overjet of 7 mm. Intraorally microdontia, maxillary anterior spacing, and hypoplastic enamel were evident (Figure 9). The soft tissues were healthy, and no caries were present. The maxillary left permanent canine (UL3) was palatally impacted and four supernumerary teeth were present radiographically (Figure 10), distal to each third permanent molars (distomolars). The orthodontic treatment plan consisted of a sectional fixed appliance to idealize the space for the impacted canine tooth and a functional appliance to reduce the overjet. Orthodontic tooth movements progressed unremarkably.

## 3 DISCUSSION

Mucopolysaccharidoses are rare lysosomal storage disorders (LSD), representing a heterogenous group of diseases arising from a deficiency in one of the eleven enzymes responsible for the breakdown of glycosaminoglycans (GAGs).^1^, ^3^ This results in their accumulation in a range of cell types inducing tissue and organ degeneration.^4^

Dental features are also dependent on the distinct type of MPS (Table 1). Identification of dental abnormalities can be of clinical significance. For example, their presence may be useful for differential diagnosis in MPS IV (Morquio Syndrome) patients. Dental findings are exclusive to MPS IVA and are absent in MPS IVB.^3^ Liaison with the metabolic team can therefore be indispensable in confirming the specific enzyme deficiency in Morquio syndrome.

**TABLE 1:** Dental features and abnormalities that are more common in MPS patients^1^, ^2^, ^3^, ^9^, ^10^

MPS Type	Dental features
All types of MPS	High caries experience, periodontal disease, delayed eruption and prolonged retention of teeth, macroglossia, thick lip, condylar defects
Hurler Syndrome (MPS I)	Gingival hyperplasia, delayed dental eruption, malocclusion, radiolucency in maxilla / mandible, condylar defects, taurodontism, hypodontia, microdontia
Hunter Syndrome (MPS II)	Gingival hyperplasia, delayed dental eruption, malocclusion, radiolucency in maxilla / mandible, condylar defects
Sanfilippo Syndrome (MPS III)	Obliteration of pulp chambers, irregular pulpal morphology
Morquio Syndrome (MPS IV)	Enamel defects (discoloration, hypoplastic), tooth surface loss, spaced dentition, peg shaped incisors, cross bites, flattened condyles
Maroteaux Lamy Syndrome (MPS VI)	Dentigerous cysts, fibrous gingival dysplasia, bony rarefactions, enlarged marrow spaces, osteosclerosis, TMJ pathology, tooth impaction, root resorption, taurodontism

As seen in the fifth case, patients with Morquio syndrome can present with enamel defects and resultant tooth surface loss in both the primary and secondary dentition.^1^ Pitted enamel was evident in our patient (Figure 9). The direct effects of deficiency of N‐acetyl‐galactosamine‐6‐sulfatase (GALNS) in Morquio A syndrome are expected to be the causative mechanism of enamel defects.^5^ Deficiency of GALNS may result in the pathological accumulation of keratin sulfate and chondroitin 6‐sulphate in the lysosomes of ameloblasts during the secretory stage of amelogenesis.^5^, ^6^ It is anticipated that glycosaminoglycans, namely keratin sulfate act as a matrix for cohesive attachment between enamel and dentine.^6^

Malocclusions can also be a presenting feature in MPS patients. Studies present a range of skeletal class I, II, and III malocclusions.^2^ Contributory etiological factors may include the propensity toward macroglossia, vertical growth patterns, hypodontia, supernumerary teeth, delayed dental eruption, and prolonged retention of teeth.^1^, ^2^ Bony pathology such as bone rarefactions, osteosclerosis, and expanded marrow spaces are present in some forms of MPS, especially Maroteaux Lamy Syndrome (MPS VI).^1^ Evidently, this could have an impact on orthodontic treatment provision.

Other dental features are described with variable frequency in the scientific literature. Cleft lip and /or palate (CLP), as seen in case one, is periodically described. The majority of these patients, however, have a contributory family history of CLP, thus making it difficult to ascertain the direct etiology and relationship with mucopolysaccharidosis. Supernumerary teeth have been reported broadly across MPS diagnoses, particularly type VI (Maroteaux‐Lamy Syndrome)^7^, ^8^ and MPS IVA (Morquio Syndrome),^3^ with supernumerary molars being the most frequently cited.

The medical management of MPS is dependent on the type (MPS I, II, III, IV, VII, and IX). Common treatment modalities fall into the broad categories of surgical intervention of MPS sequelae, enzyme replacement therapy, hematopoietic stem cell transplantation and conservative management. Hematopoietic stem cell transplantation for MPS I is most effective when given to young children (<2 years). In these patients, the dental team should remain vigilant to the collateral effects that potential chemotherapy and radiotherapy have on nutrition and dental development and adapt their therapeutic approach accordingly. Enzyme replacement therapy provides a valuable treatment modality in many subtypes of MPS (I, II, IV, VI). Given intravenously, challenges in crossing the blood brain barrier (BBB) limit its use to MPS subtypes with widespread neurological symptoms, such as Sanfilippo Syndrome. Vimizim, a type of enzyme replacement therapy (ERT) is one treatment modality for MPS IVA and was the treatment of choice for our patient.

Mucopolysaccharidoses present a plethora of physical and psychological manifestations. GAG deposition is both chronic and progressive thus having profound effects on numerous bodily systems. Cardiovascular sequelae can be substantial, ranging from conduction defects, valvular disease, cardiomyopathy, and progressively to heart failure and considerable pulmonary hypertension in some patients.^9^ Neurological manifestations are also variable and dependent on the MPS type. Profound neurological symptoms are most apparent in Sanfilippo Syndrome (MPS III), where progressive behavioral disturbances are common.

The management of MPS patients with gross caries therefore presents an additional challenge for clinicians. The intellectual disability and neurological symptoms that present in several types of MPS often necessitates dental intervention under general anesthetic.^1^, ^4^ This can produce significant challenges for the anesthetist and varying levels of risk. Airway management may be compromised in MPS patients due to the susceptibility for glycosaminoglycan accumulation in the head and neck region.^2^ Subsequent adenotonsillar hypertrophy, airway narrowing, soft tissue thickening, and macroglossia can occur.^9^ Atlantoaxial instability and odontoid hypoplasia are also frequent features.^9^ Systemically, the anesthetist must consider any underlying cardiac comorbidity or obstructive airway disease.

The potential challenges to behavioral management under local anesthetic compel an aggressive approach to caries prevention. Fastidious management is therefore fundamental in such patients to safeguard against the elevated anesthetic risk. Regular fluoride varnish application, liaison with their metabolic dietician (where relevant), intensive oral hygiene instruction and early identification of dental caries and infection are essential.

## CONFLICTS OF INTEREST

None declared.

## AUTHOR CONTRIBUTION

LH selected the cases and wrote the manuscript. SM reviewed, edited, and provided guidance for subsequent drafts of the manuscript. AC verified and contributed to the metabolic content of the manuscript. GAA was the treating clinician for 4 of the cases and provided guidance for the content of the manuscript.

## ETHICAL APPROVAL

Informed written consent was obtained from all parents in this case series prior to submission regarding the publication of images and data.

## CONSENT

Informed verbal and written consent was obtained from all parents / legal guardians of all cases. The purpose of the case series was explained to all participants including what information would be published. All participants were advised that declining consent would not impact their clinical care and that they have the right to withdraw their consent at any point prior to publication.

## Data Availability

Data sharing was not applicable to this article as no datasets were generated or analyzed in the production of the manuscript.
